# I wish someone had told me …

**Published:** 2011-09

**Authors:** Karinya Lewis

**Affiliations:** Registrar, Royal Bournemouth Hospital Eye Unit, Castle Lane East, Bournemouth, Dorset BH7 7DW, UK

**Figure:**
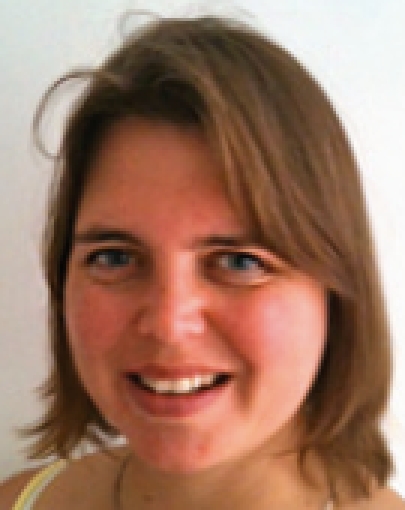

**Key learning points**

- The greatest risk factor for loss of vision due to diabetic retinopathy is failure to attend for retinal screening or treatment.
- Many people with diabetes are not aware that diabetes may affect their eyes and may not understand why screening and early treatment are required.
- Education of diabetes patients is the responsibility of everyone involved in their care — don't assume someone else will do it.
- When providing laser treatment, be sympathetic and explain that it may be painful, but that you will take the utmost care to make the patient as comfortable as possible.
- Everyone with dibetes should be taught the ‘Things every diabetes patient should know’ (see box).

Regular retinal examinations are essential for identifying diabetic retinopathy and applying timely laser treatment before significant vision is lost. This is known as retinal screening and is a key part of the care of all diabetes patients. However, non-attendance at eye clinic for screening has been recognised as an independent risk factor for poor visual outcome from diabetic retinopathy.^1^

In 2005, we undertook a qualitative study involving focus groups and interviews in a rural and urban area to identify some of the reasons why patients fail to attend for diabetic eye examinations.^2^ Although our study was conducted in the United Kingdom (UK), many of the findings will be very relevant to people with diabetes wherever they live. Because the UK provides free health care for all citizens, the cost of treatment was not a barrier in this study, but it is likely to be a major barrier where patients have to pay for an eye examination and for laser treatment.

We categorised the barriers into three main areas:

- Patient beliefs
- Social attitudes
- Enabling and disabling factors.

## Patient beliefs

Even though most UK patients knew that diabetes could affect their eyes, many were not aware that it could lead to severe visual impairment and blindness. In part, this was because health workers were very reluctant to use the word ‘blindness.’ In addition, patients did not realise that they would only experience symptoms once DR had become very advanced, and that treatment would be most effective if given before there were symptoms. Some patients with type 2 diabetes thought that it was ‘less severe’ than type 1 diabetes, and that they were therefore unlikely to experience problems with their vision. As a result, they thought screening was unnecessary.

Patients' expectations of laser treatment were high and they hoped that their vision would improve. They were therefore often disappointed in the outcomes; this led to loss of confidence in the health services and, later, non-attendance. Laser treatment was frequently described as both painful and frightening for patients. Doctors were perceived as unsympathetic, which also made patients less likely to return.

Patients who failed to attend reported that they were afraid of finding out how bad their retinopathy was. Some patients were aware that progression of retinopathy was linked to poor blood sugar control. However, many did not understand that it is inevitable to develop some retinopathy after having diabetes for twenty years, no matter how well it is controlled. When these patients were referred to the eye clinic, they felt that this was because they had failed to control their diabetes, giving rise to feelings of guilt and low self-esteem.

## Social attitudes

Hospitals were seen as places for the sick, so for patients who seemed well, hospital attendance for regular retinal screening was not seen as ‘normal behaviour.’ Whilst most employers seemed to be willing to give time off for occasional appointments, time off for regular appointments was not tolerated. Patients felt that, ultimately, it would cost them their jobs.

Patients with diabetes have multiple hospital appointments, most of which are for routine surveillance, and some patients chose to attend only the appointments they saw as essential. Often, this meant they did not attend for retinal screening.

Those patients with poor family support, or whose relatives had a limited understanding of the disease, were less likely to attend for eye examinations, as this was not a priority for the family as a whole.

## Enabling and disabling factors

In the rural area, transport to and from the clinic was a major barrier as there is poor public transport. Patients with their own vehicles were not permitted to drive due to the dilating eye drops. In the urban area, this was less of an issue as patients were willing and able to use public transport.

Clinic waiting times were identified as a barrier by both patients and providers. Patients said that the delays made them reluctant to ask questions of the doctor to help them better understand their DR. It also made it difficult for friends or relatives to accompany patients or provide transport.

**Things every diabetes patient should know**

- Diabetes will eventually affect the blood vessels in your eyes. This is called diabetic retinopathy, and it can lead to visual impairment and blindness.
- By controlling your blood sugar and blood pressure, you can reduce the damage diabetes can cause in your eyes. However, your eyes will eventually develop some diabetic changes. If you do get diabetic retinopathy, it is not your fault.
- Diabetic retinopathy in most people has no symptoms — you cannot tell if you have it. Only an examination of the back of your eyes can find it, and you should be examined every year.
- Diabetic retinopathy is treatable if it is found in the early stages. If you attend all your screening or clinic appointments, and have treatment when recommended, it is very unlikely that you will go blind.
- If you do not attend diabetic eye screening or eye clinic appointments, your diabetic retinopathy can become very advanced and will affect your vision. If left untreated, you may go blind.
- Modern treatments with laser and drugs are very effective in stopping vision loss. However, treatment cannot restore vision that has already been lost.

**Figure:**
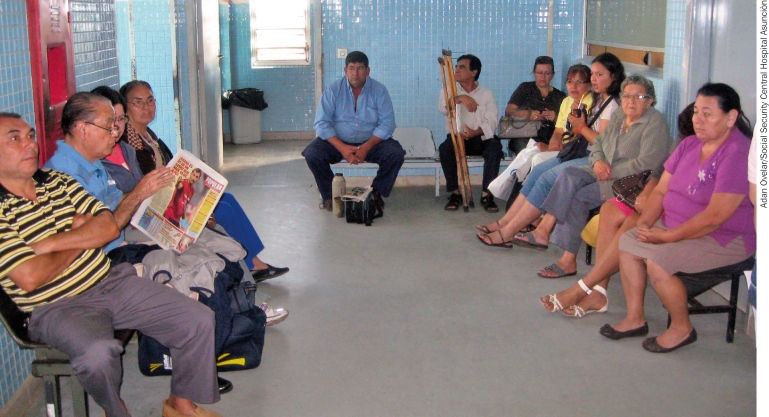
Long waiting times in the clinic can make some patients reluctant to come for a retinal examination. PARAGUAY

## Global perspectives

Our study found that the commonest barrier was a lack of awareness about diabetic eye disease and its treatment; this is consistent with other studies. As retinal screening programmes have become more common in low- and middle-income countries, preliminary research^3^ from China, Zambia, Qatar, and Paraguay have all consistently shown that, fundamentally, a lack awareness of the severity of diabetic eye disease and a need for preventive treatment before symptoms develop are the main reasons why patients do not access eye care services.

- The study in Zambia, where diabetic eye care was just being established, showed that most patients knew nothing about eye complications as a result of diabetes. They were struggling with much more basic issues such as how to monitor blood sugar.
- The study in China found that a quarter of patients presented for the first time with advanced diabetic retinopathy, and the only variable independently associated with late presentation was lower education level.
- In Paraguay and Pakistan, where programmes have existed for longer, lack of awareness still remained the primary barrier but was closely followed by difficulty in accessing services, more so in rural areas than urban areas.
- Research carried out in Qatar, an Arabic state, highlighted how social attitudes to women can lead to different barriers in accessing eye care services. For men, the barriers were ‘too busy’ and ‘no appointments’; for women, they were ‘too great a distance to travel alone’ or ‘lack of transport’.

The next most commonly reported barrier is the cost of consultation and laser treatment,^4^ which will always be an issue where screening and treatment are not subsidised.

## Tackling the different barriers

### Patient beliefs

It appears that personal beliefs and attitudes to diabetic eye disease are similar across the world and are best tackled through appropriately educating the patient. Eye care providers must play a key role in teaching patients about diabetic eye disease (see opposite). Do not assume that other health providers will have done so. Information about diabetic eye disease, screening, and treatment needs to be accurate, informative, and yet non-intimidating. It is often better received if it is tailored to the individual. Patients seem to benefit from seeing the changes in their own retinal images, where this is possible.

### Social attitudes

In areas where social and cultural beliefs about diabetes or eye care services are detrimental to health seeking behaviour, mass media or marketing campaigns may be the best strategy. Patients are most influenced by their family, so including family members in teaching sessions, consultations, and decisions is key to changing patients' behaviour.

### Enabling and disabling factors

The purpose of retinal screening is to prevent loss of vision, particularly for those people who are less likely to seek out health services. Screening services therefore need to be made as accessible as possible. Common barriers for health professionals to address may include: patient transport, clinic delays, limited and inconvenient timing of appointments, insufficient notice or publicity, intimidating health staff, and painful laser treatment.

Patients will not attend diabetic eye care programmes if they don't understand why it is important and when it is appropriate. As health care providers we have to ensure that correct information about DR and its treatment is communicated effectively to the patient and their family. If patients come for screening, we must make the patient's experience as convenient, efficient, helpful, and painless as possible to ensure that they return regularly. These barriers are possible to overcome and are sight saving.
